# Enantioconvergent catalysis

**DOI:** 10.3762/bjoc.12.192

**Published:** 2016-09-16

**Authors:** Justin T Mohr, Jared T Moore, Brian M Stoltz

**Affiliations:** 1 Department of Chemistry, University of Illinois at Chicago, 845 West Taylor St., Chicago, IL 60607, USA; 2 The Warren and Katharine Schlinger Laboratory for Chemistry and Chemical Engineering, Division of Chemistry and Chemical Engineering California Institute of Technology, 1200 E California Blvd. MC 101-20, Pasadena, CA 91125, USA, Fax: (+1) 626-395-8436

**Keywords:** asymmetric catalysis, enantioselectivity, synthetic methods

## Abstract

An enantioconvergent catalytic process has the potential to convert a racemic starting material to a single highly enantioenriched product with a maximum yield of 100%. Three mechanistically distinct approaches to effecting enantioconvergent catalysis are identified, and recent examples of each are highlighted. These processes are compared to related, non-enantioconvergent methods.

## Review

**Figure 1:**
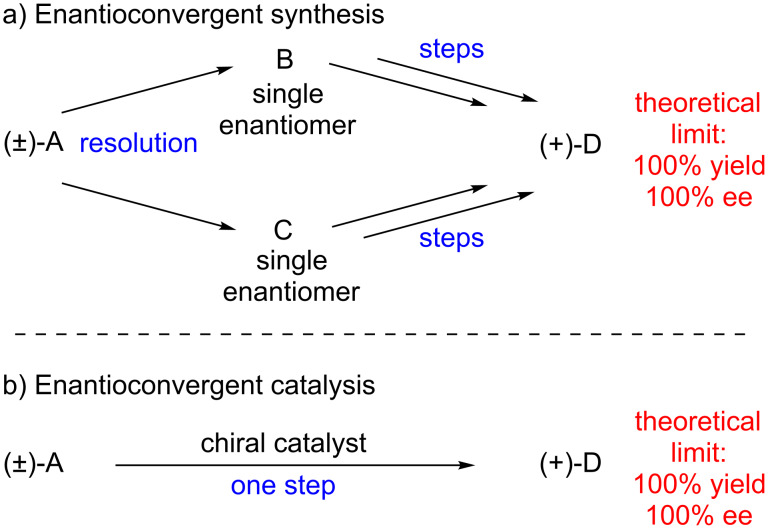
Enantioconvergent methods.

Enantioconvergent synthetic sequences are powerful methods which convert a racemic starting material to a highly enantioenriched product in up to 100% yield (Figure 1a) [1]. These routes circumvent the inefficiency inherent to many traditional enantioselective reactions with racemic materials (e.g., kinetic and classical resolution), which generally have a maximum chemical yield of 50%. Enantioconvergent synthesis requires partitioning the synthetic pathway into two distinct sequences, and then merging the materials to the same product and is therefore a compromise in terms of step economy [2]. Despite the additional synthetic operations required, enantioconvergent synthesis has seen broad applications in the construction of complex molecules [3–5].

A more efficient strategy is to employ a single catalytic asymmetric transformation capable of converting a racemate directly to a highly enantioenriched product in high chemical yield (Figure 1b). Noyori has stated that this complex transformation is an “ideal asymmetric catalysis” [6]. There are several potential challenges in designing such a reaction, including kinetic resolution of the starting material and double stereodifferentiation [7] of intermediates. Despite these potential pitfalls, significant advances toward this goal have been realized. Examples of several of these successes are identified in this review.

Enantioconvergent catalytic processes can be classified according to differences in their mechanistic pathways and the hypothesized reactive intermediates. Three important types of enantioconvergent catalysis are specifically discussed herein: type I – stereomutative, type II – stereoablative [8], and type III – parallel kinetic resolution [9]. The primary criteria for all enantioconvergent catalytic reactions are:

1. The starting material must be racemic.
2. A catalyst must be involved in the reaction process and induce the asymmetry in the product.
3. The product must be isolated in enantioenriched form.
4. Each antipode of the racemic starting material must lead to the same major enantiomer of product.

### Type I: Stereomutative enantioconvergent catalysis

**Figure 2:**
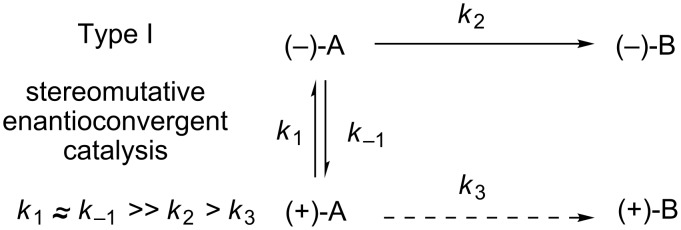
Stereomutative enantioconvergent catalysis.

Type I (stereomutative) enantioconvergent catalysis typically involves two distinct catalytic cycles: the first performs a rapid equilibration between the two enantiomers of the racemic starting material – a process known as stereomutation – while the second cycle selectively converts one enantiomer to product (Figure 2). Additionally, the rate of starting material racemization must be significantly faster than the rate of kinetic resolution in order to achieve maximum yield and selectivity.

**Scheme 1:**
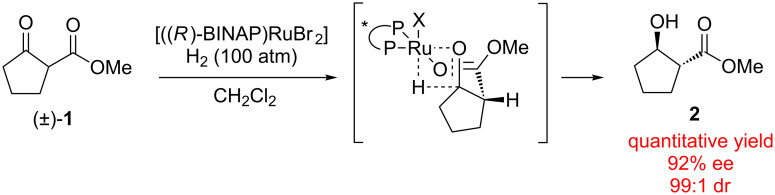
Dynamic kinetic resolution by hydrogenation.

Perhaps the most well-developed class of type I enantioconvergent catalysis is dynamic kinetic resolution (DKR) [10–16]. Processes of this type were first described by Noyori in the enantioselective hydrogenation of β-ketoesters ((±)-**1** → **2**, Scheme 1) [6]. In the event, the resident stereocenter of the substrate **1** can epimerize via tautomerization to the enol form. Deuterium labeling experiments have shown that the hydrogenation reaction occurs only on the chiral keto tautomer, and therefore the catalyst selects one enantiomer of the substrate when the reduction takes place.

**Scheme 2:**
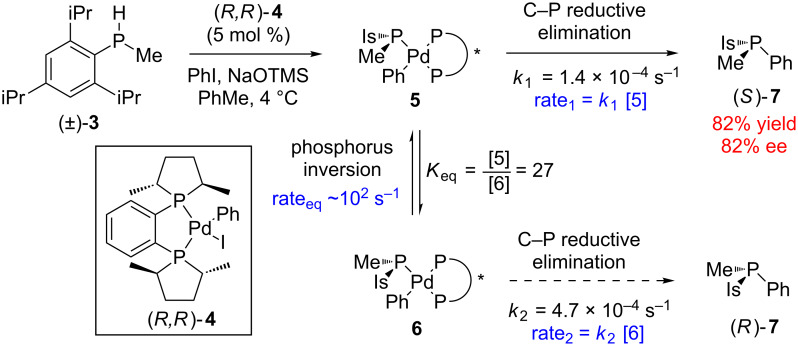
Enantioconvergent synthesis of phosphines governed by Curtin–Hammett/Winstein–Holness kinetics (TMS = trimethylsilyl, Is = 2,4,6-triisopropylphenyl).

Enantioconvergent methods are not limited to carbon stereocenters. An exceptional example of type I enantioconvergent catalysis was reported by Glueck for the preparation of enantioenriched *P*-chiral phosphines (Scheme 2) [17]. In this process, enantiopure Pd complex (*R*,*R*)-**4** reacts with racemic phosphine **3** to form phosphido complexes **5** and **6**. Although the rate of configurational inversion of these two complexes was not observed directly for this system, extrapolation from related systems gives a rate more than 10^5^ times greater than the observable rates of C–P reductive elimination. Since the rate of inversion is much greater than the rate of bond formation, Curtin–Hammett/Winstein–Holness kinetics [18] were employed to elucidate to the overall process. Interestingly, although the observed rate constants (*k*_1_ and *k*_2_) indicate that bond formation occurs more rapidly from complex **6** (leading to (*R*)-**7**), the equilibrium strongly favors the diastereomeric intermediate **5**, and the corresponding difference in concentration leads to a greater observed rate for the formation of (*S*)-**7** (i.e., rate_1_ > rate_2_). Notably, if this stereomutation pathway were absent (the case of simple kinetic resolution) then the relative rate difference between the two complexes would fall outside the typical range considered synthetically useful for synthesis. This example specifically highlights the importance of relative reaction rates in stereomutative enantioconvergent catalysis.

### Type II: Stereoablative enantioconvergent catalysis

**Figure 3:**
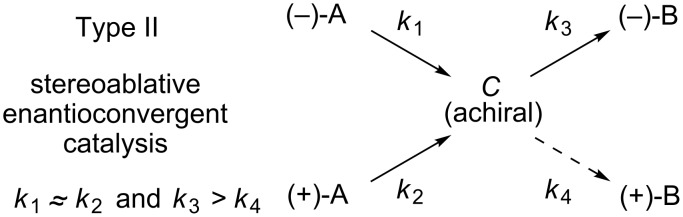
Stereoablative enantioconvergent catalysis.

Type II (stereoablative [8]) enantioconvergent catalysis (Figure 3) is composed of processes in which a racemic starting material is irreversibly transformed into an achiral intermediate that subsequently undergoes an enantioselective conversion to the product. Reports of this type are predominantly in the areas of prochiral enolates and prochiral metal π-allyl complexes [19–21]. Critical to the success of such a method is a comparable rate of reaction for the two components of the racemate with respect to the stereoablative mechanistic step (i.e., *k*_1_ ≈ *k*_2_, Figure 3). If this condition is not met, significant kinetic resolution will occur, causing product yield to suffer. Additionally, there must be a significant difference in the rates of product formation (i.e. *k*_3_ > *k*_4_). If this condition is not met, enantioselectivity will suffer.

**Scheme 3:**
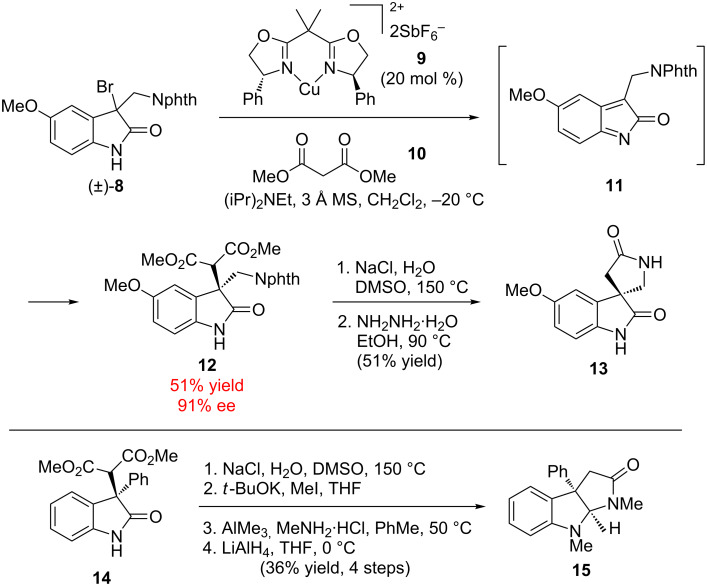
Stoltz’ stereoablative oxindole functionalization.

Stoltz and co-workers have reported an approach for the preparation of enantioenriched oxindole derivatives from racemic oxindole halides using a stereoablative approach (Scheme 3) [22–23]. Deprotonation and elimination of the halide in oxindole (±)-**8** leads to achiral azaxylylene intermediate **11**, which is trapped with malonate nucleophiles to form all-carbon quaternary centers. The overall transformation is unusual since oxindoles are typically nucleophilic, but in this case the stereoablative step in the mechanism leads to an electrophilic intermediate. The use of Cu(box) complex **9** rendered the reaction enantioselective, forming C-3 quaternary oxindole **12** in 91% ee (up to 94% ee for related substrates). This strategy is useful for constructing spiro- and fused-pyrrolidinoxindole architectures, such as lactam **13** and aminal **15**, found in several natural product families. Related approaches with organic catalysts were explored in 2012 by Yuan and co-workers [24–25] and in 2014 with tertiary amine squaramide catalysis by Lou and co-workers [26].

**Scheme 4:**
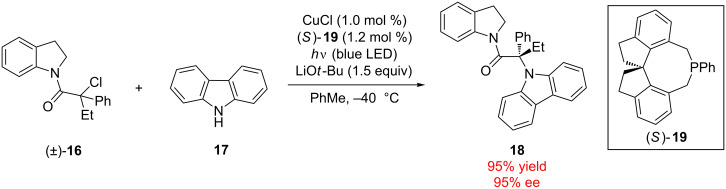
Fu’s type II enantioconvergent Cu-catalyzed photoredox reaction.

The generation of radical intermediates from chiral sp^3^-hybridized halides presents another opportunity for type II enantioconvergent catalysis. Peters and Fu have reported a system for the Cu-catalyzed C–N cross-coupling of racemic tertiary alkyl halide electrophiles with carbazole nucleophiles induced by visible light (Scheme 4) [27]. Although the mechanism continues to be studied, it is hypothesized that irradiation of the copper–carbazole complex leads to an excited-state adduct that is capable of generating achiral tertiary alkyl radical intermediates through electron transfer with a racemic alkyl halide (e.g., (±)-**16**). Subsequently, the achiral radical combines with the chiral Cu catalyst and undergoes an enantioselective bond-formation step in conjunction with the carbazole nucleophile to form α-aminoamide **18**. This report fuses both enantioconvergent and photoredox catalysis, two powerful and modern methods. A similar strategy was employed by Fu and MacMillan in 2016 [28].

**Scheme 5:**
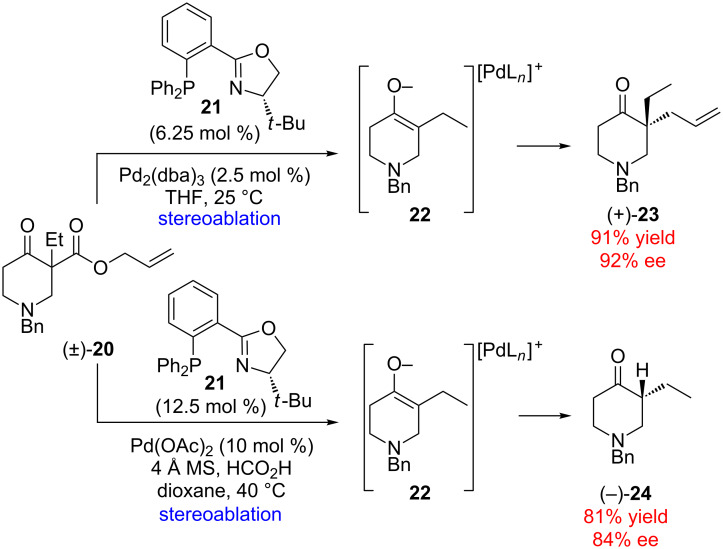
Stereoablative enantioconvergent allylation and protonation (dba = dibenzylideneacetone).

Type II enantioconvergent catalysis is especially powerful when a single reagent effects both the stereoablative (typically bond-breaking) and stereoselective (bond-forming) steps of the process. An example of such a reaction was reported by Stoltz for the generation of enantioenriched all-carbon quaternary stereocenters from racemic allyl β-ketoesters (e.g., (±)-**20** → (+)-**23**, Scheme 5) [29]. This particular reaction is especially unusual since the stereoablative step requires scission of a C–C bond at a quaternary carbon stereocenter to form achiral enolate intermediate **22**. Since no kinetic resolution of the racemic starting material was observed, yields in excess of 90% with up to 92% ee could be obtained.

In further studies, it was found that the putative enolate intermediate could also be trapped by a proton source to yield α-tertiary cycloalkanones in high ee (e.g., (±)-**20** → (–)-**24**) [30]. Interestingly, in the reactions of certain substrates the enolate face functionalized by the electrophilic allyl group is opposite to the face functionalized by the proton (Scheme 5). This observation indicates that the two enantioconvergent reactions, though related, must proceed through substantially different mechanisms of enantioinduction. The differential reactivity demonstrated by the enolate intermediate **22** highlights the power of accessing different mechanistic pathways via stereoablative initiation.

**Scheme 6:**
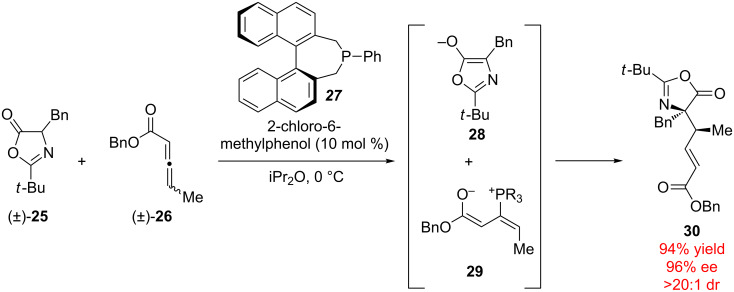
Enantioconvergent allylic alkylation with two racemic starting materials.

Examples with multiple racemic starting materials are rare since each additional racemic substrate exponentially increases the number of stereochemical combinations. However, Kalek and Fu have demonstrated that racemic allenoates (±)-**26** and racemic azalactones (±)-**25** may be combined in the presence of an enantiopure phosphine catalyst **27** in order to generate the coupled product **30** with both high ee and dr (Scheme 6) [31]. Presumably, the allenyl stereochemistry is destroyed upon 1,4-addition of the phosphine catalyst, resulting in chiral phosphonium adduct **29** that further reacts with deprotonated oxazole **28**. The resulting intermediate undergoes proton transfer and elimination of the phosphonium moiety, resulting in product **30** and regeneration of the catalyst. This exceptional demonstration of stereocontrol requires that the catalysts precisely organize both the electrophilic and nucleophilic reactants to control the formation of asymmetric carbons on each fragment. The doubly stereoconvergent nature of this reaction represents one of the most complex examples of stereoablative enantioconvergent catalysis to date.

### Type III: Enantioconvergent parallel kinetic resolution

**Figure 4:**
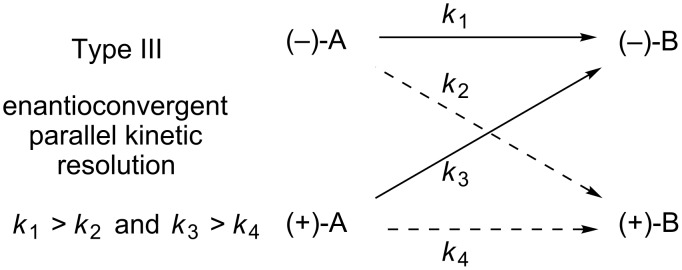
Enantioconvergent parallel kinetic resolution.

A third approach to enantioconvergent catalysis is depicted in Figure 4. Similar to type I, reactions of this type involve a kinetic resolution of the racemic starting material. However, in this case the two enantiomers undergo separate modes of reactivity, each leading to an identical product. Reactions of this type are variants of the powerful parallel kinetic resolution (PKR) strategy [32–33], owing to the two parallel processes occurring simultaneously. Although PKR reactions significantly increase the observed product ee relative to a simple kinetic resolution system, the theoretical maximum yield for a traditional PKR is still limited to 50%. In contrast, an enantioconvergent PKR process allows formation of enantiopure materials in up to 100% yield.

**Scheme 7:**
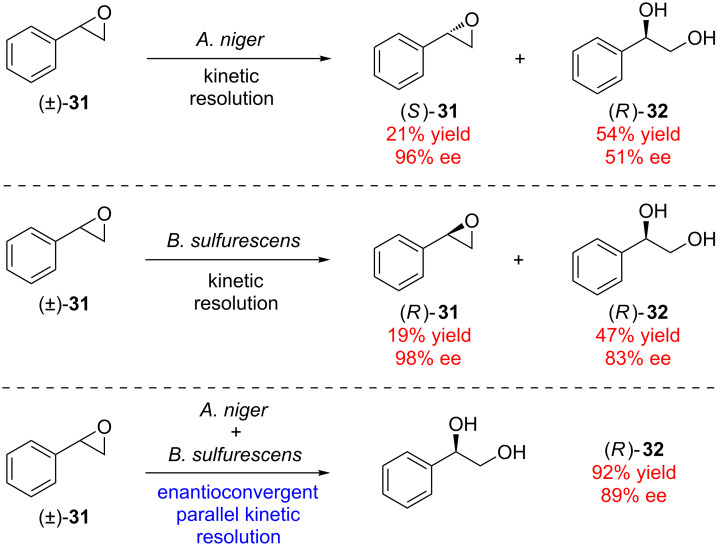
Enantioconvergent parallel kinetic resolution by two complementary biocatalysts.

Although examples of enantioconvergent PKR are rare, some biocatalysts have succeeded in effecting this unusual transformation [34]. Furstoss found that two microorganisms (*Aspergillus niger* and *Beauveria sulfurescens*) were capable of resolving racemic styrene oxide (**31**, Scheme 7) by hydrolytic kinetic resolution [35]. It was recognized that these two biocatalysts exhibited opposite enantiomer preference in the kinetic resolution event. Moreover, the major hydrolysis byproduct **32** of each of these kinetic resolutions had the same absolute configuration. Combining these two complementary catalysts leads to a highly efficient parallel process wherein each catalyst enantioselectively hydrolyzes one enantiomer of the epoxide, ultimately forming diol (*R*)-**32** in 92% yield with 89% ee [36].

**Scheme 8:**
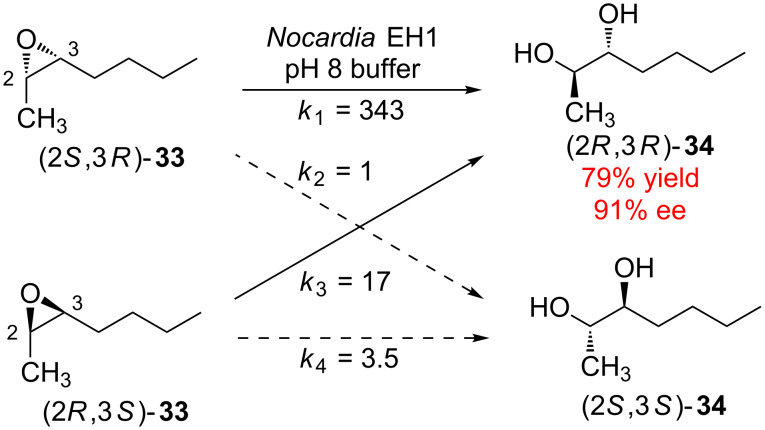
Enantioconvergent PKR by *Nocardia* EH1.

An especially remarkable example of type III enantioconvergent catalysis utilizes a single enzymatic catalyst. Faber observed that *Nocardia* EH1 is capable of catalyzing the hydrolysis of racemic epoxide **33** to the corresponding diol (2*R*,3*R*)-**34** in 79% chemical yield with 91% ee (Scheme 8) [37]. The observed product arises from hydrolysis of each enantiomer of epoxide at the *S*-configured carbon atom. Isotopic labeling studies with ^18^OH_2_ not only confirmed this mechanistic hypothesis, but also facilitated kinetic studies to determine relative rate constants for each of the four reaction pathways (*k*_1_−*k*_4_, Scheme 8). It was found that (2*S*,3*R*)-**33** hydrolyzes rapidly (*k*_1_ = 343) with preference for addition at C(2), forming (2*R*,3*R*)-**34**. Hydrolysis of the enantiomeric epoxide occurs selectively at C(3) (*k*_3_ = 17), which also leads to (2*R*,3*R*)-**34**. Interestingly, kinetic resolution of the starting material occurs with modest selectivity relative to many enzymatic processes (*k*_rel_ = (*k*_1_ + *k*_2_)/(*k*_3_ + *k*_4_) = 17). In fact, for optimal performance in PKR, it is desirable to obtain a similar rate of reaction for the two enantiomers of the starting material in order to maintain the ideal 1:1 substrate ratio and maximize the selectivity [29]. While the typical kinetic resolution suffers from a decline in product ee at >50% conversion, the enantioconvergent nature of this process maintains high enantiopurity even at very high conversion. To date, purely chemical methods of catalysis that rival these interesting type III transformations are limited and represent a challenge to the field.

## Conclusion

Enantioconvergent catalysis is a powerful method for the efficient construction of enantiopure materials for a variety of synthetic uses. Although these transformations are often complicated by unfavorable double stereodifferentiation, the recent appearance of several mechanistically unique methods to address this problem is indicative of a bright future for this chemistry. As demonstrated by the examples in this review, precise understanding of the kinetic factors at play in a reaction is critical to its success. Continuing development in this field may lead to the “ideal asymmetric catalysis” [3].
